# Neuraminidase Subtyping of Avian Influenza Viruses with PrimerHunter-Designed Primers and Quadruplicate Primer Pools

**DOI:** 10.1371/journal.pone.0081842

**Published:** 2013-11-29

**Authors:** Yanyan Huang, Mazhar Khan, Ion I. Măndoiu

**Affiliations:** 1 Department of Pathobiology and Veterinary Science, University of Connecticut, Storrs, Connecticut, United States of America; 2 Department of Biology, Memorial University of Newfoundland, St. John’s, Newfoundland and Labrador, Canada; 3 Department of Computer Science and Engineering, University of Connecticut, Storrs, Connecticut, United States of America; National Institute for Viral Disease Control and Prevention, CDC, China, China

## Abstract

We have previously developed a software package called PrimerHunter to design primers for PCR-based virus subtyping. In this study, 9 pairs of primers were designed with PrimerHunter and successfully used to differentiate the 9 neuraminidase (NA) genes of avian influenza viruses (AIVs) in multiple PCR-based assays. Furthermore, primer pools were designed and successfully used to decrease the number of reactions needed for NA subtyping from 9 to 4. The quadruplicate primer-pool method is cost-saving, and was shown to be suitable for the NA subtyping of both cultured AIVs and uncultured AIV swab samples. The primers selected for this study showed excellent sensitivity and specificity in NA subtyping by RT-PCR, SYBR green-based Real-time PCR and Real-time RT-PCR methods. AIV RNA of 2 to 200 copies (varied by NA subtypes) could be detected by these reactions. No unspecific amplification was displayed when detecting RNAs of other avian infectious viruses such as Infectious bronchitis virus, Infectious bursal disease virus and Newcastle disease virus. In summary, this study introduced several sensitive and specific PCR-based assays for NA subtyping of AIVs and also validated again the effectiveness of the PrimerHunter tool for the design of subtyping primers.

## Introduction

Avian Influenza Viruses (AIVs) are a large group of eight-segmented, negative-sense RNA viruses belonging to the family Orthomyxoviridae. Wild aquatic birds are the natural reservoir of AIVs [1,2]. According to the genetic and antigenic difference of the two surface glycoproteins, hemagglutinin (HA) and neuraminidase (NA), AIVs are separated into different subtypes. To date, 16 HA and 9 NA subtypes of AIVs have been detected from wild birds [3,4]. Human AIV infections of multiple subtypes have been previously reported [5-7]. Global-scale epidemiological surveillance of AIVs in wild birds is reinforced in the last two decades to track virus evolution and prepare ourselves for a possible Avian Flu Pandemic [4,8-10]. 

Current strategy of AIV surveillance is to firstly screen the swab samples by Matrix-protein gene Real-time RT-PCR (RRT-PCR) [11]. The positive samples are then identified for their HA and NA subtypes. Whole AIV genome sequences may be further determined depending on the purpose of a study [4,12]. Therefore, a sensitive, specific and time-saving subtyping method will greatly facilitate the AIV epidemiological surveillance. The traditional method for NA subtyping is to perform neuraminidase inhibition (NI) assays with cultured AIVs, which is also the gold standard for AIV diagnosis suggested by World Health Organization [13]. However, NI assays are time-consuming and can only be performed in labs possessing HA and NA anti-serum panels. PCR-based molecular diagnostic tests such as RT-PCR, Real-time PCR (R-PCR), Real-time RT-PCR (RRT-PCR) are more efficient approaches for AIV subtyping, and better suited for high throughput sample analysis during AIV surveillance [14]. Some prior studies have focused on the identification of a small number of featured NA subtypes, such as N1 and N2 [15,16]. The increasing NA sequences of multiple subtypes available in NCBI flu database makes it possible to design primers for all NA subtyping [17]. A one-step RT-PCR assay with degenerate primers followed by sequencing was reported in 2008 to subtype different NA subtypes [18]. Since 2009, several RT-PCR methods were reported for NA gene subtyping [19-23]. 

We have previously developed an open source software package called PrimerHunter for the design and selection of virus-subtyping primers. Different from the primer-design software employed by the above reports, PrimerHunter ensures the amplification specificity by accurately estimating the melting temperature with mismatches, computed based on the nearest-neighbor model. The advantage of PrimerHunter was confirmed by the successful HA subtyping of AIVs in Real-time PCR (R-PCR) [24]. In this study, NA-subtyping primers were designed and validated for their applicability with several PCR methods. Additionally, we used powerful integer programming techniques in conjunction with primer-dimer prediction tools to design pools of PrimerHunter primers that can be used to reduce the number of PCR reactions from 9 to 4, decreasing cost and increasing detection sensitivity.

## Materials and Methods

### Virus strains

AIVs of all 9 NA subtypes were provided by United States Department of Agriculture (USDA), which are A/TY/MA/40550/87-Bel/42 (H5N1), A/TY/CA/209092/02 (H5N2), A/TY/CA/35621/84 (H5N3), A/TY/Ontario/6118/67 (H8N4), A/DK/Alberta/60/76 (H12N5), A/Mallard/Alb/331/85 (H3N6), A/Waterfowl/GA/269432-56/03 (H5N7), A/TY/Ontario/63 (H6N8) and A/TY/WI/68 (H5N9). Several other avian pathogens such as avian infectious bronchitis virus (IBV, Massachusetts 41strain), avian infectious bursal disease virus (IBDV, D78 strain) and Newcastle disease Virus (NDV, Lasota strain) were maintained in our lab and are used for specificity tests of the assays.

### Design of NA-subtyping primers

NA subtype-specific primers were designed with PrimerHunter as previously described [24]. A total of 668 complete NA sequences of AIVs in North America (as of March 2008) were downloaded from the NCBI flu database. Primer Hunter was run once for each subtype. When designing primers for a certain NA subtype (Ni) we used all available NA sequences of the subtype (Ni) as targets, and all NA sequences of other subtypes (non-Ni) as non-targets. Between 7 and 9,665 pairs of primers were designed by PrimerHunter for each NA subtype (complete primer lists are available at http://dna.engr.uconn.edu/software/PrimerHunter/ ). One pair of primers for each NA subtype was selected for further experiments. The primer sequences used in this study and the expected PCR lengths are shown in Table 1.

**Table 1:** Primer sequences designed for NA subtyping and the expected PCR products.

NA subtype	Primer sequence	Product length (bp)^*+*^	TM value^*++*^
N1-foward	5'-TAGACTGCATGAGGCCTTGCTTCTG-3'	137	78.2~79.2
N1-reverse	5'-CACCGTCTGGCCAAGACCAACCTAC-3'		
N2-foward	5'-ATGTTATCAATTTGCACTTGGGCAG-3'	149	77.1-77.8
N2-reverse	5'-CATGCTATGCACACTTGTTTGGTTC-3'		
N3-foward	5'-ATGATGTCTCTTGGACAAGCAATAG-3'	104	74.8-76.2
N3-reverse	5'-TGGGCATAAACCCAATGTTGGAACC-3'		
N4-foward	5'-AAATCATAACCATCGGTAGTGCGAG-3'	194	76.8-78.6
N4-reverse	5'-TATAGTTGTTCTGCACATTGGTGAC-3'		
N5-foward	5'-CATTTGTGGCATGTGGTCCCACGGA-3'	147	76.6-77.2
N5-reverse	5'-AGGCATTGGGTGAAGATCCTAATGG-3'		
N6-foward	5'-GCAAATAGACCAGTAATCACTAT-3'	153	77.9-78.9
N6-reverse	5'-CCAGGATCTGGGTTTCCTCCTGTTA-3'		
N7-foward	5'-AGCCAAGTATGTTTGGTGGACGAGC-3'	111	79.2-80.3
N7-reverse	5'-TTACGAAAAGTATTGGATTTGTGCC-3'		
N8-foward	5'-TAATGAGTGTAGAAATAGGGCAATC-3'	127	78.9-79.6
N8-reverse	5'-GGAATCAGGGCCCGTTACTCCAA-3'		
N9-foward	5'-ATCGTATTAAACACTGACTGGAGTG-3'	171	78.2-78.9
N9-reverse	5'-ATTCTGTGCTGGAACACATTGATAC-3'		

+ The expected length of PCR product was calculated according to reference sequences of corresponding NA subtype in NCBI influenza database.

++ The range of TM value was analyzed by serially diluted DNA or RNA template of different NA subtype in Real-time PCR or Real-time RT-PCR.

### Sensitivity and specificity of the NA-subtyping primers in Real-time PCR

#### RNA extraction, amplification and cloning of NA genes

Viral RNAs of the 9 NA subtypes were extracted from AIV-containing allantoic fluids with Trizol LS Reagent (Invitrogen, USA). One-step RT-PCRs were carried out with each of the 9 primer pairs according to the manufacturer’s protocol (Qiagen, USA, One-step RT-PCR kit). The reaction mixture (25 μl volume) contains: 14μl nuclease free water, 5μl Qiagen one-step RT-PCR buffer, 1μl Enzyme mix, 1μl dNTP mix, 2μl plasmid DNA, 1μl (0.3-0.6μM) of each primer. The RT-PCR conditions were 50°C for 30min, 95°C for 15min, 40 cycles of 95°C for 30s, 40°C for 30s and 72°C for 1min, followed by 72°C for 10min at the end of the reaction.

PCR products of the expected lengths were purified by QIAquick PCR purification kit (Qiagen, USA), and then cloned with TOPO TA cloning kit (Invitrogen, USA). Plasmids were extracted (Wizard® Plus SV Minipreps DNA Purification System, Promega) and identified by PCR method, and then confirmed by DNA sequencing (DNA Biotechnology Facility, UCONN). The copy number of the NA gene-plasmids was calculated according to the formula: copies/ul = (The plasmid’s concentration × 6 × 10^14^) / (The plasmid’s base pairs × 660). 

#### Real-time PCR

Plasmids of the 9 NA subtypes (10^5^ copies) worked as the templates for R-PCR to test the specificity of the designed primers. R-PCR was performed in triplicate with Power SYBR Green PCR Master Mix (ABI, USA). The reaction mixture (25 μl volume) contains: 8.5μl nuclease free water, 12.5μl PCR Master, 2μl plasmid DNA, 1μl (0.3-0.6μM) of each primer. The R-PCR conditions were 95°C for 10min, 40 cycles of 95°C for 15s, 40°C for 30s and 60°C for 1min, followed by dissociation curve analysis R-PCR amplification and detection were conducted with the ABI 7500 system. The data of Ct value, Tm value and the product output (as reflected by the derivative of the dissociation curve) were systematically analyzed using the 7500 system SDS software (version 1.4). Gel electrophoresis was performed to confirm the size and singularity of the products after R-PCR. 

In order to validate the applicability of the primers in the NA subtyping of AIVs with varied concentrations, N1, N2, N3 and N7-specific primers were tested in R-PCR with each of the serially diluted on- and off-target plasmids (1 to 10^8^ copies) as template. The amplification data were analyzed according to our previous report [24]. In brief, when the fluorescent signal is not detectable (e.g. in a NTC reaction), the Ct value is set to 40. For each reaction, ΔCt is computed as the difference between the Ct of NTC reactions and the Ct of each reaction.

### Sensitivity of the NA-subtyping primers in Real-time RT-PCRs

#### In vitro transcribed NA genes

The 9 NA plasmids were linearized using restriction enzyme-BamHI (Promega, USA). The linearization products were then visualized by gel eletrophoresis and purified using Qiaquick PCR Purification Kit (Qiagen, USA). RNAs were synthesized in vitro from the linearized NA plasmids with RiboMAX™ Large Scale RNA Production Systems-T7 kit (Promega, USA).

#### Real-time RT-PCR

Serially diluted RNA standards of each NA subtype (from in vitro transcription), with copy numbers ranging from 1 to 10^10^, were performed RRT-PCR with corresponding primers following the protocol of Power SYBR Green RNA-to-CT 1-Step kit (ABI, USA). Detection limits of RRT-PCR to each NA subtype were determined. The reaction volume is 20μl, containing 1μl standard RNA template and 0.3-0.6μM of each primer. The RRT-PCR amplification consisted of 48°C for 30min (reverse transcription), 95°C for 10min, followed by 40 cycles of 95°C for 15 seconds, 40°C for 30 seconds and 60°C for 1 minutes. Fluorescence was measured at the end of incubation at 60°C. The results of amplification were analyzed as mentioned above.

### Design of primer pools for NA subtyping

#### Principle of primer pool design

To reduce the number of reactions needed to identify the 9 NA subtypes, primer pairs of more than one NA subtypes must be used in one R-PCR reaction. Clearly, primer pools must be designed so that the result of these reactions uniquely identifies the subtype present in the sample. After the selection of a detection threshold, the result of each reaction can be viewed as being either positive or negative (in practice, additional information is provided by dissociation curves in R-PCR or RRT-PCR). Thus, primer pools must be designed so that each subtype results in a unique pattern of positive and negative signals, which can be viewed as the “barcode” for the subtype. Ensuring unambiguous identification is equivalent to ensuring that for each pair of subtypes there is a pool that results in a positive signal for one but not for the other [25]. We additionally require that each subtype results in positive amplification signal from at least one pool. There are several additional constraints that we should take into account when designing pools of primer pairs. First, pool size cannot be too large, since this will result in decreased amplification efficiency due to the reduced primer concentration. We will denote by “2m” the maximum number of primers allowed in a primer pool. Second, primer-dimers must be avoided since even a single pair of dimerizing primers can lead to complete loss of amplification signal. 

#### Procedure of primer pool design

We tested all primers included in Table 1 using the online autodimer tool at the National Institute of Standards and Technology (http://yellow.nist.gov:8444/dnaAnalysis/primerToolsPage .do ). With a total score threshold of 4, autodimer reported 10 potentially dimerizing primer pairs. In general, we denote the set of pairs of subtypes whose primers are predicted to form dimers by D. No dimerizing pair of primers in *D* can be included in the same primer pool without compromising detection accuracy. Additionally, primer pools must be designed in such a way that the resulting PCR amplification pattern uniquely identifies the virus subtype. Subject to these constraints, we would like to minimize the number of pools, i.e., the number of PCR reactions. Pool design can be modeled as an integer linear program similar to that proposed by Rash and Gusfield for DNA barcoding [25]. The minimum number of uniquely decodable pools subject to primer non-dimerization constraints can then be found by solving the integer program using either freely available solvers such as GNU GLPK (https://www.gnu.org/software/glpk/) or commercial solvers such as the IBM CPLEX optimizer (http://www-01.ibm.com/software/commerce/optimization/cplex-optimizer/). Let N denote the number of subtypes, identified for simplicity by the integers from 1 to N (N = 9 in the case of NA subtyping). We first construct the set *P* of candidate pools, i.e., subsets of size at most *m* of {1, . . . ,N} that do not contain any pair in D. We then introduce a 0/1 variable *x*_*p*_ for every candidate poolp∈P, where *x*_*p*_ is set to 1 if pool p is selected in our subtyping assay and to 0 otherwise. The pool design problem can then be written as follows: 

minimize $\sum_{p\in P}^{}x_{p}$ (1)

s.t. $\sum_{p\in P:|\{i,j\}\cap p|=1}xp\geq 1,\;\;1\leq i<j\leq N$

$$\begin{array}{c} \sum_{p\in P:i\in p}xp\geq 1,\;\;1\leq i\leq N\\ xp\in \{0,1\},\;\;p\in P \end{array}$$

To break ties in favor of solutions that use pools with fewer primers, objective (1) can be replaced by 

minimize $\sum_{p\in P}^{}\left(M+|P|\right)x_{p}$ (2)

where M is a constant larger than |P|N. For NA subtyping, this results in an integer program with 85 variables (pool candidates) and 45 constraints that was solved to optimality in a fraction of a second using CPLEX.

The resulting assay consisting of 4 pools is shown in Table 2 along with expected amplification signatures for each NA subtype.

**Table 2:** Quadruplicate primer pools for NA subtyping.

Primer pool (NA subtypes)	NA subtypes of AIVs								
	N1	N2	N3	N4	N5	N6	N7	N8	N9
A (N2、N6、N7)		+				+	+		
B (N4、N5、N7、N8)				+	+		+	+	
C (N3、N5、N9)			+		+				+
D (N1、N4、N6、N9)	+			+		+			+

Primer pools were obtained by solving integer program (2) using the IBM CPLEX optimizer. Notice that each column corresponds to a unique amplification pattern, which allows unambiguous identification of the NA subtype present in the sample.

### Specificity of the pooled-primers in Real-time PCRs

The quadruplicate R-PCR reactions (A, B, C and D) are expected to give distinct results to different subtypes of NA genes. Primer-pool R-PCR (pR-PCR) was carried out with each of the 9 NA plasmids (10^5^ copies) as template to validate the specificity of the pooled-primers for NA subtyping. 

### Specificity of the primer-pool in Real-time RT-PCRs

One-step RRT-PCR (Power SYBR® Green RNA-to-CT™ 1-Step kit, ABI, USA) was performed with the primer pools (pRRT-PCRs) and the in vitro-transcribed RNA (10^5^ copies) as template. The reaction volume and amplification cycles are the same to those in RRT-PCR with individual NA primer pair (see above). The result of each reaction (positive or negative) is determined by the analysis of Ct value, derivative of dissociation curve and the TM value. RNA of each NA subtype extracted from cultured AIVs were also tested by the pRRT-PCRs. Specificity of the assays was also determined with pRRT-PCR by detecting RNAs of several other avian pathogens, such as IBV, IBDV and NDV. The reaction system and RRT-PCR cycle parameters are the same as described above.

### Application of the primers in NA subtyping of AIV swab samples

Since 2010, the NA subtyping primers in Table 1 have been employed to determine the subtypes of AIV swab samples from wild birds (ducks, gulls and murres) at St. John’s, Canada. Amplification of longer NA sequences was attempted, after NA subtyping, either with other primers, or with the combination of other primers with the NA subtyping primers in this study. The acquired NA sequences were used to confirm the accuracy of the NA subtyping methods. P-value computation based on a simple multinomial i.i.d. null model was performed to analyze the accuracy of PCR-based NA subtype assignments. Briefly, assuming independence between samples, the probability of an ordered sequence of subtype assignments *y*_*1*_, *y*_*2*_, …, *y*_*n*_ was computed as ∏_i=1,…,n_ P(*y*_*i*_) [26]. For every sample *i*, the probability of assigning subtype *k* was assumed to follow a multinomial distribution in which the probability of a subtype is given by its frequency according to sequencing based assignments, namely 3/65 for N8 and N9, 4/65 for N1, N3, and N4, 18/65 for N6, and 29/65 for N2; probabilities of all remaining subtypes were assumed to be zero.

## Results

### Sensitivity and specificity of Real-time PCRs with individual NA-subtyping primer pairs

**Figure 1:**
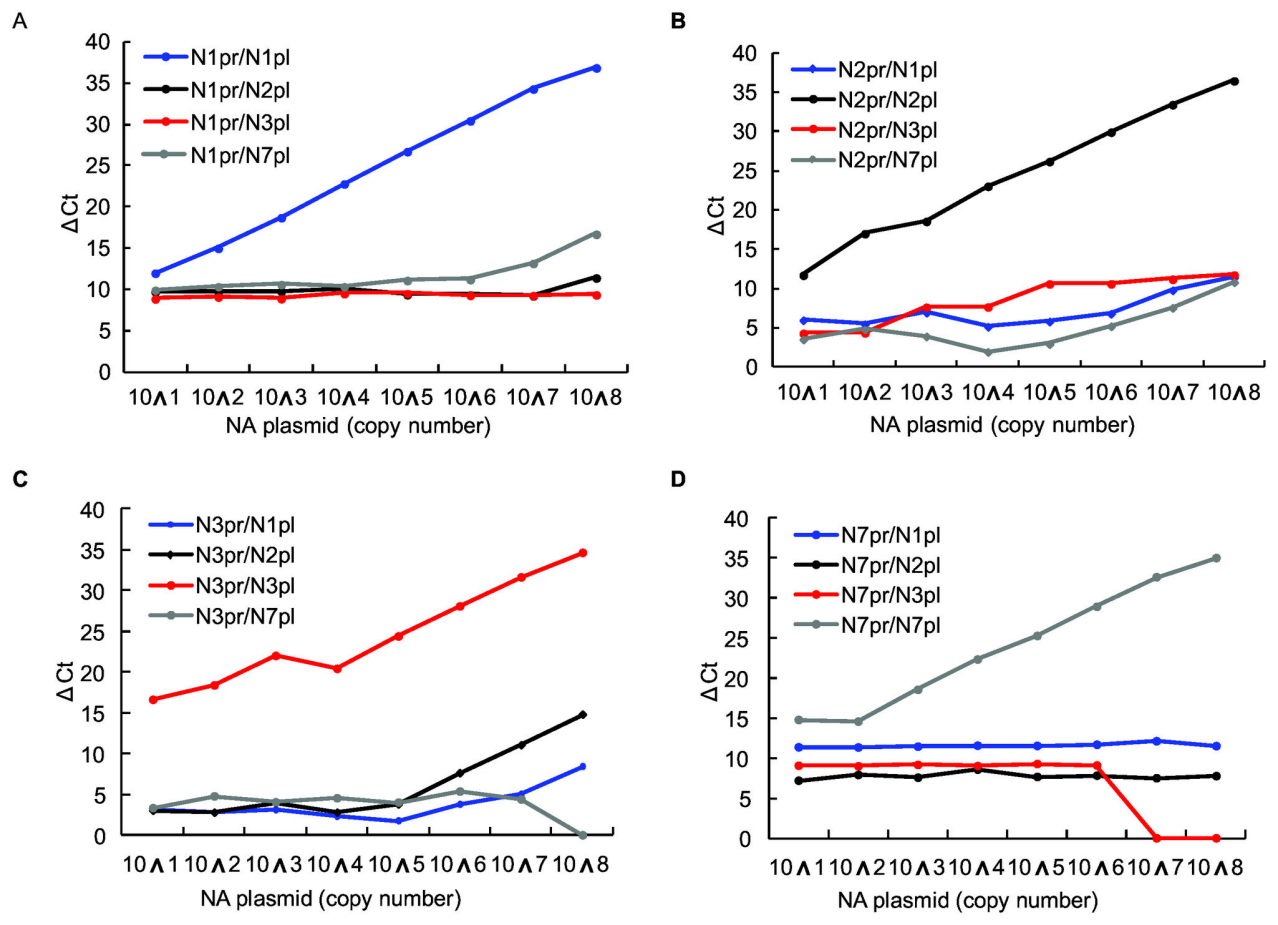
Specificity of the NA-subtyping primers in Real-time PCRs. Real-time PCRs were performed with N1, N2, N3 and N7-specific primer pairs and 10-fold serially diluted on- and off target NA plasmid templates (10 to 10^8^ copies), the ΔCt values for them were recorded in A, B, C and D part of Figure 1, respectively. "N1pr/N1pl" represents Real-time PCR with N1-plasmid template and N1-specific primers, and so on and so forth.

All primer pairs only reacted with their respective NA subtypes and showed excellent specificity in R-PCR. TM values of R-PCR products of all NA subtypes were listed in Table 1. Figure 1 showed the ΔCt of the R-PCRs performed with N1, N2, N3 and N7-specific primers. The plasmids of each NA-subtype with wide concentration range showed exponential amplification in these reactions. Taking Ct=30 as the cut-off value, the R-PCRs could perform NA subtyping of 1~10 copies (1 copy of N3 and N7 genes, 10 copies of N1 and N2 genes, respectively). Positive reactions should have Ct≤30 plus expected Tm-value range (as shown in Table 1) and regular dissociation curve.

### Sensitivity of Real-time RT-PCRs with individual NA-subtyping primer pairs

One-step RRT-PCR with in vitro-transcribed RNA as template showed exponential amplification of each NA subtype with RNA of 1 to 10^10^ copies per reaction (Table S1). Besides the Ct value (≤30), positive reactions should have expected Tm-value range (Table 1) and regular dissociation curve. The developed RRT-PCRs could perform NA subtyping for AIV RNAs of 2~200 copies (depending on the subtype). The sensitivity of the assays varies by NA subtype as shown in Table S1.

### Results of Real-time PCR with primer pools

**Figure 2:**
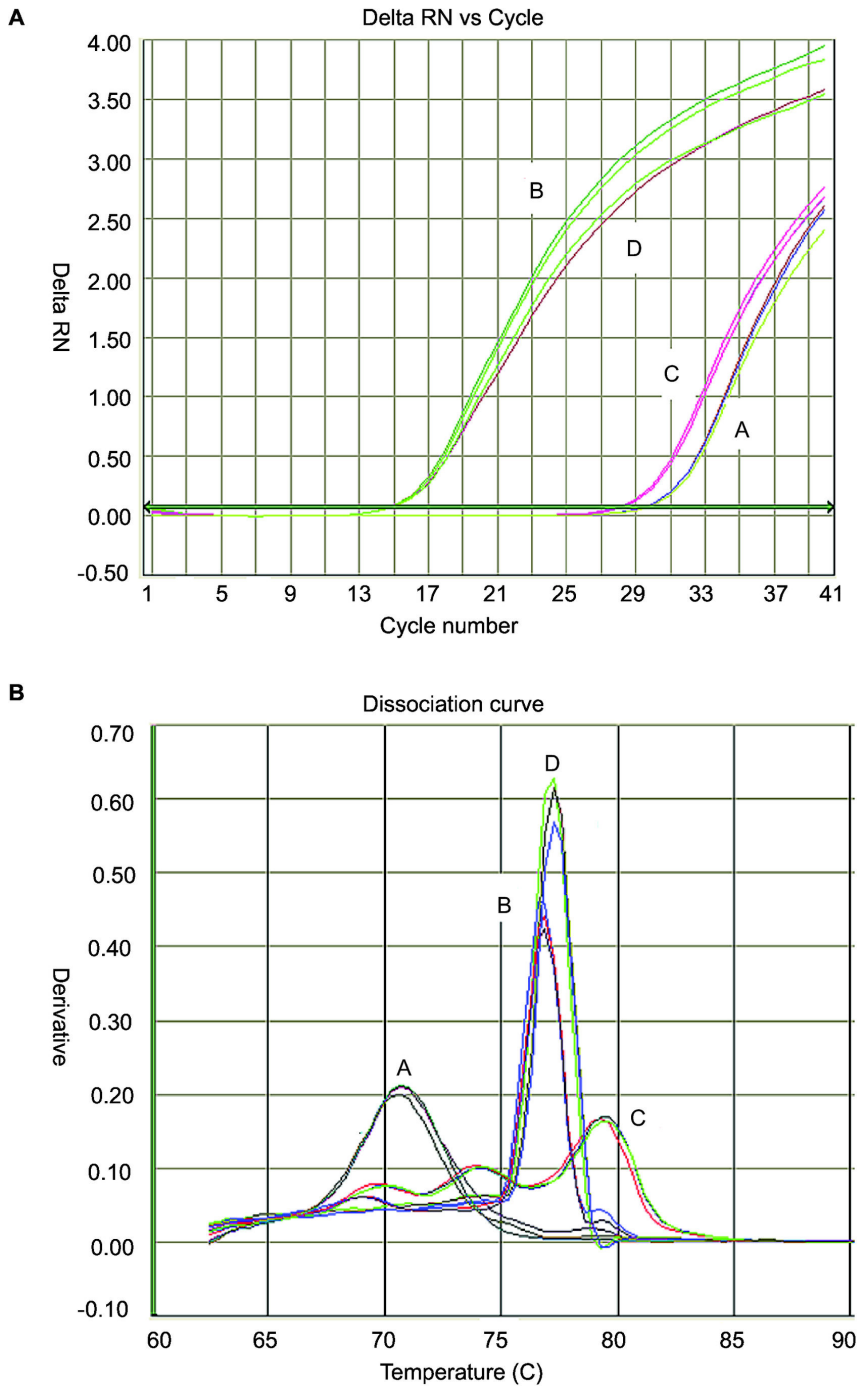
Results of primer-pool Real-time PCR with N4-subtype plasmid as the template. The amplification curves (part A of Figure 2) showed robust amplification (positive results) of B and D reactions and weak ones (negative results) of A and C reactions. The dissociation curves of B and D reactions are distinct from those of A and C reactions.

The amplification results of each NA plasmid by R-PCRs using the 4 primer pools were as expected. All of the 9 NA-specific primers selectively reacted with corresponding NA plasmid (Table S2). The amplification properties (measured by slopes of amplification curve and Ct values) of the 4 reactions with primer pools for all NA plasmids were similar to those with individual NA-specific primers. The result of each reaction (positive or negative) is determined by the analysis of Ct value, derivative of dissociation curve and the TM value as mentioned above. Ct value of 28 was primarily chosen as the cut-off value of amplification. The positive reaction should have Ct≤28 with expected Tm-value range (as shown in Table 1) and dissociation curve. Taking the primer-pool test of the N4 plasmid as an example, the amplification curves in Figure 2 showed positive amplification of B and D reactions, but negative results for A and C reactions. The dissociation curve of the four reactions also validated the robust amplification of B and D reactions with expected ranges of the Tm values. 

### Results of Real-time RT-PCR with primer pools

One-step RRT-PCR with in vitro-transcribed RNA and primer pool (pRRT-PCR) showed similar results as primer pooled R-PCR (Table S3). A Ct value of 28 was preliminary chosen as the threshold of pRRT-PCR. Additionally, a positive reaction should have the expected Tm-value range of the NA subtype (Table 1) and the regular dissociation curve. RNA of each NA subtype extracted from AIV-containing allantoid fluids of specific-pathogen-free (SPF) embryonated chicken eggs showed specific amplification in pRRT-PCRs. Taking again the N4-subtype AIV as example, the N4 RNA displayed similar amplification curves and dissociation properties in pRRT-PCR (Figure S2) as tested by primer-pool R-PCR with N4-subtype plasmids (Figure 2). No amplification curves were displayed when RNA of IBV, IBDV or NDV was used with pRRT-PCRs, demonstrating the specificity of the reactions to AIV NA genes. 

### Results of the NA subtyping of uncultured AIV samples

With the subtype-specific primers, 33 duck AIVs (4 N1 subtypes, 10 N2 subtypes, 1 N3 subtype, 4 N4 subtype, 9 N6 subtypes, 4 N8 subtypes and 1 N9 subtype), 14 gull AIVs (3 N3 subtypes, 9 N6 subtypes and 2 N9 subtypes) and 21 murre AIVs (all N2 subtypes) in St. John’s have their NA subtypes determined as shown in Table S4. Either single primer-pair RT-PCR or primer-pool RT-PCR was used to determine the NA subtypes of the 68 AIVs during these experiments. The NA subtypes of 65 viruses were confirmed by gene sequencing, and the results showed coincidence with the subtyping results by RT-PCR (Table S4). Sequencing data was available for 42 of the 45 samples tested by single primer pair RT-PCR, and all 42 samples had concordant subtypes assigned by the two methods (p-value 7.04593e-21). All 23 samples tested by primer-pool RT-PCR had sequencing data available, and all of them had concordant subtypes (p-value 2.53858e-23, p-values were computed assuming a multinomial i.i.d. null model with subtype frequencies given by the empirically observed frequencies in the sequenced samples). Primer-pool RT-PCR and sequencing results for 5 of the clinical AIV samples are given in Figure S1. 

## Discussion

This study validated the applicability of PrimerHunter in designing virus-subtyping primers and also introduced several sensitive and specific methods for the subtyping of NA genes of AIVs. Different from most primer design software packages, PrimerHunter takes both target and non-target sequences as input sets for primer design and ensures the specificity of PCR amplification by relying on accurate melting temperature computations with mismatches [24]. Different PCR methods introduced in this study provided multiple choices for NA subtyping depending on the availability of corresponding PCR facilities. The 9 NA subtyping primer pairs showed excellent sensitivity and specificity in R-PCR with the template of NA plasmids, in RRT-PCR with in vitro transcribed NA-gene RNA as the template, and also in RT-PCR with RNAs of AIV swab samples. The quadruplicate primer pools developed in this study are also a good choice for NA subtyping. 

The determination of the results for R-PCR and RRT-PCR in this study is facilitated by the differential TM values and dissociation curves of different NA subtypes, as primers leading to PCR products of differential TM values were selected [27]. It is especially helpful in primer pooled R-PCR and RRT-PCR, since multiple primer combinations used in a single reaction may increase the chance of unspecific amplification. PrimerHunter can ensure the designed primer pairs specific to a certain subtype do not dimerize, but the dimerization may occur between primer pairs of inappropriate combination in pooled-primer PCR reactions, which happened to our initial design of the primer pools. Plentiful efforts were used to screen and validate (through experiments) the specific primer combinations in this study. The primer that caused unspecific reactions was detected by experiments and after the selection of a new primer and the adjustment of the primer-pool combination, satisfactory results were acquired in pR-PCR and pRRT-PCR. 

The quadruplicate primer-pool methods in this study reduces costs and labor for NA subtyping by decreasing the number of reactions required to differentiate NA subtypes from 9 to 4, therefore, when it comes to the NA subtyping of samples with limited RNA quantity, the primer pool test could relatively improve the sensitivity of the detection by adding more RNA sample in each of the 4 reactions, compared to the performance of 9 separate reactions. This method is especially valuable when dealing with uncultured AIV samples, such as the bird swab samples collected during AIV epidemiological surveillance. The subtyping of these samples is frequently constrained by both the limited volume of samples and low concentration of their RNAs. When dealing with AIV (oral or fecal) swab samples, it is necessary to pre-treat the AIV samples by centrifuging and filtration (0.45-μm membrane) methods to get rid of the possible bacterium contamination, since bacteria may cause unspecific reactions in the NA subtyping (proved by DNA sequencing in our experiment). Although the product lengths of the contaminations are usually different from those of the NA genes in RT-PCR in our study, it is easier to identify the results with pure and specific amplifications. It should be noted that the pooled-primer subtyping method cannot differentiate the NA subtypes of mixed-AIV infections. However, the method could still reduce the reactions needed to differentiate the involved NA subtypes, by analyzing the related NA subtypes in positive reactions and the annealing temperature of the PCR products. The suspected NA genes can then be further tested with individual NA-subtype primer pairs. 

In summary, the designed NA subtyping primers and the developed PCR methods in this study can be used to subtype cultured AIVs, and are also suitable for NA subtyping of uncultured AIV-positive samples during large-scale AIV surveillance.

## Supporting Information

Table S1 **Sensitivity tests of Real-time RT-PCR for N1 to N9 RNA.** Serially diluted RNA standards (1 to 10^10^ copies) for each NA subtype were performed Real-Time RT-PCR (RRT-PCR) with corresponding primers. Mean Ct value and Tm value were calculated for the 3 repetition of each test. Positive reactions have Ct≤30 with expected Tm-value range (as shown in Table 1) and regular dissociation curve (even and single-peak curve indicating the robust amplification of a single product).
(DOC)

Table S2 **Result of Real-time PCR with pooled primers for N1 to N9 plasmid.** NA plasmids (10^5^ copies) of each NA subtype were used as the templates for Real-time PCR. The primer-pool combination comprises 4 reactions, A, B, C and D, as showed in Table 2. Mean Ct value and Tm value were calculated for the 3 repetition of each reaction. "-" means Tm value of the R-PCR product is too low to be detected or the dissociation curve (DC) is irregular. Positive reactions have Ct≤28 with expected Tm-value range (as shown in Table 1) and dissociation curve.
(DOC)

Table S3 **Result of Real-time RT-PCR with pooled primers for N1 to N9 RNA.** Pooled primers and the RNA templates of each NA subtype extracted from AIV-infected allantoid fluids were used in Real-time RT-PCR. The primer-pool combination comprises 4 reactions (A, B, C and D) as shown in Table 2. Mean Ct value and Tm value were calculated for the 3 repetition of each reaction. The symbol "-" means the Tm value of the R-PCR product is too low to be detected or the dissociation curve (DC) is irregular (e.g. multiple peaks). Positive reactions have Ct≤28 with expected Tm-value range (as shown in Table 1) and dissociation curve.
(DOC)

Table S4 **NA subtypes of the 68 AIV swab samples determined in this study.** Sixty-eight AIV swab samples from wild birds (ducks, gulls and murres) at St. John’s, Canada were determined for their NA subtypes by RT-PCR with the primer pairs designed in this study. Subsequent amplification and sequencing of longer NA genes were performed, either with other primers, or with the combination of other primers with the NA-subtyping primers in this study, to confirm the accuracy of the NA subtyping methods in this study. The Genbank accession numbers of the NA genes were available for 57 of the 68 samples. *represents samples (23) determined for their NA subtypes by primer-pool RT-PCRs, while other samples (45) were performed individual primer paired RT-PCRs.
(DOC)

Figure S1 **Gel electrophoresis image of one-step RT-PCR with pooled primers.** The electrophoresis was run with 1.5% agarose gel. A, B, C and D represent pooled-primer reaction A to D, as showed in Table 2. M represents 100bp DNA marker (NEB, USA). The NA-subtyping results for the 5 swab samples were further confirmed by gene sequencing and related genes were submitted to the Genbank database under accession numbers KC464568, KC492344, KC464592, KC492256, and KC492368.
(DOC)

Figure S2 **Result of Real-time RT-PCR with pooled primers and N4 RNA template.** RNA was extracted from H8N4 AIV-infected allantoid fluids. The amplification curves (part A) showed positive amplification in B and D reactions of the primer-pooled Real-time RT-PCR, while negatives in A and C reactions. The dissociation curves (part B) of B and D reactions are distinct from those of A and C reactions.
(DOCX)
