# Correction: Neuraminidase Subtyping of Avian Influenza Viruses with PrimerHunter-Designed Primers and Quadruplicate Primer Pools

**DOI:** 10.1371/annotation/0c03da4e-c4f6-4992-8c5b-bdabdd3bb08f

**Published:** 2013-12-19

**Authors:** Yanyan Huang, Mazhar I. Khan, Ion Măndoiu

The name of the second author is incorrect. The correct name is: Mazhar I. Khan. The correct abbreviation for this author's name in in the Author Contributions statement is: MIK. The name of the third author is also incorrect. The correct name is: Ion Măndoiu. The correct abbreviation in the Author Contributions statement for this author's name is: IM. The correct Citation is: Huang Y, Khan MI, Măndoiu I (2013) Neuraminidase Subtyping of Avian Influenza Viruses with PrimerHunter-Designed Primers and Quadruplicate Primer Pools. PLoS ONE 8(11): e81842. doi:10.1371/journal.pone.0081842. 

