# Supplementary material for: Neuraminidase Subtyping of Avian Influenza Viruses with PrimerHunter-Designed Primers and Quadruplicate Primer Pools
Source: PLoS One. 2013 Nov 29;8(11):e81842. doi: 10.1371/journal.pone.0081842 (PMC3843705; doi:10.1371/journal.pone.0081842)
Supplement: Table S1 — Sensitivity tests of Real-time RT-PCR for N1 to N9 RNA. Serially diluted RNA standards (1 to 1010 copies) for each NA subtype were performed Real-Time RT-PCR (RRT-PCR) with corresponding primers. Mean Ct value and Tm value were calculated for the 3 repetition of each test. Positive reactions have Ct≤30 with expected Tm-value range (as shown in Table 1) and regular dissociation curve (even and single-peak curve indicating the robust amplification of a single product). (DOC) [file pone.0081842.s001.doc]

**Table S1. Sensitivity tests of Real-Time RT-PCR for N1 to N9 RNA.**

Serially diluted RNA standards (1 to 10^10^ copies) for each NA subtype were performed Real-Time RT-PCR (RRT-PCR) with corresponding primers, and the results were shown in Table S1 from A to I. Mean Ct value and Tm value were calculated for the 3 repetition of each test. The detection limit of the RRT-PCR for each NA subtype was determined, and the Ct value of 30 was set as the cut-off value of the reaction. The higher the value of derivative of dissociation curve (DC), the more robust the amplification is. Positive reactions should have Ct≤30 with expected Tm-value range (as shown in Table 1) and regular dissociation curve (even and single-peak curve indicating the robust amplification of a single product).

**Table S1-A** RRT-PCR results for serially-diluted RNA of N1 subtype

| X (RNA copies=20.8×10^x^) | Mean Ct value | Derivative of DC (≤) | Mean Tm value |
| --- | --- | --- | --- |
| 1 | 29.29147 | 0.37 | 78.1 |
| 2 | 25.45415 | 0.45 | 78.5 |
| 3 | 23.02127 | 0.48 | 78.43 |
| 4 | 19.6078 | 0.51 | 78.33 |
| 5 | 16.2164 | 0.54 | 78.5 |
| 6 | 12.6112 | 0.60 | 78.38 |
| 7 | 9.027677 | 0.60 | 78.43 |
| 8 | 5.63306 | 0.60 | 78.43 |
| 9 | 3.710983 | 0.58 | 78.43 |
| NTC | - | - | - |

**Table S1-B** RRT-PCR results for serially-diluted RNA of N2 subtype

| X (RNA copies=14.4×10^x^) | Mean Ct value | Derivative of DC (≤) | Mean Tm value |
| --- | --- | --- | --- |
| 0 | 34.74 | 0.16-0.28 | 77.3 |
| 1 | 29.53835 | 0.40 | 77.35 |
| 2 | 25.42097 | 0.48 | 77.4 |
| 3 | 23.97113 | 0.50 | 77.4 |
| 4 | 21.15963 | 0.52 | 77.43 |
| 5 | 18.05117 | 0.58 | 77.63 |
| 6 | 14.85343 | 0.60 | 77.63 |
| 7 | 11.30867 | 0.59 | 77.63 |
| 8 | 8.319787 | 0.58 | 77.4 |
| NTC | 36.8786 | 0.24 | 77.53333 |

**Table S1-C** RRT-PCR results for serially-diluted RNA of N3 subtype

| X (RNA copies=4×10^x^) | Mean Ct value | Derivative of DC (≤) | Mean Tm value |
| --- | --- | --- | --- |
| 1 | 29.69225 | 0.29 | 75.75 |
| 2 | 27.5972 | 0.33 | 75.8 |
| 3 | 22.4197 | 0.36 | 75.8 |
| 4 | 18.49363 | 0.36 | 75.8 |
| 5 | 14.58393 | 0.40 | 75.8 |
| 6 | 11.8764 | 0.42 | 75.8 |
| 7 | 8.3484 | 0.43 | 75.8 |
| 8 | 5.183427 | 0.41 | 75.8 |
| NTC | - | - | - |

**Table S1-D** RRT-PCR results for serially-diluted RNA of N4 subtype

| X (RNA copies=17.54×10^x^) | Mean Ct value | Derivative of DC (≤) | Mean Tm value |
| --- | --- | --- | --- |
| 0 | 29.92513 | 0.38 | 76.96667 |
| 1 | 25.9611 | 0.45 | 76.95 |
| 2 | 21.74923 | 0.51 | 77.1 |
| 3 | 17.72903 | 0.58 | 77.1 |
| 4 | 13.72727 | 0.60 | 77.15 |
| 5 | 10.1206 | 0.66 | 77.2 |
| 6 | 6.376603 | 0.68 | 77.2 |
| 7 | 3.740117 | 0.70 | 77.1 |
| 8 | 《3 | 0.67 | 77.1 |
| NTC | - | - | - |

**Table S1-E** RRT-PCR results for serially-diluted RNA of N5 subtype

| X (RNA copies=21.6×10^x^) | Mean Ct value | Derivative of DC (≤) | Mean Tm value |
| --- | --- | --- | --- |
| 0 | 30.3529 | 0.42 | 76.43 |
| 1 | 28 | 0.45 | 76.5 |
| 2 | 24.1149 | 0.50 | 76.57 |
| 3 | 22.8081 | 0.52 | 76.57 |
| 4 | 22.004 | 0.55 | 76.72 |
| 5 | 17.0799 | 0.60 | 76.67 |
| 6 | 13.8449 | 0.60 | 76.8 |
| 7 | 10.8913 | 0.60 | 76.67 |
| 8 | 7.33045 | 0.59 | 76.67 |
| NTC | - | - | - |

**Table S1-F** RRT-PCR results for serially-diluted RNA of N6 subtype

| X (RNA copies=0.8×10^x^) | Mean Ct value | Derivative of DC (≤) | Mean Tm value |
| --- | --- | --- | --- |
| 0 | 30.46217 | 0.32 | 78.1 |
| 1 | 27.174 | 0.38 | 78.2 |
| 2 | 24.0641 | 0.42 | 78.2 |
| 3 | 21.11547 | 0.45 | 78.2 |
| 4 | 19.03677 | 0.45 | 78.2 |
| 5 | 16.34 | 0.50 | 78.2 |
| 6 | 13.447 | 0.50 | 78.2 |
| 7 | 10.547 | 0.55 | 78.2 |
| 8 | 7.64 | 0.58 | 78.2 |
| NTC | - | - | - |

**Table S1-G** RRT-PCR results for serially-diluted RNA of N7 subtype

| X (RNA copies=0.48×10^x^) | Mean Ct value | Derivative of DC (≤) | Mean Tm value |
| --- | --- | --- | --- |
| 0 | 30.8861 | 0.28 | 79.66667 |
| 1 | 27.4683 | 0.33 | 79.85 |
| 2 | 22.6127 | 0.37 | 79.9 |
| 3 | 20.31437 | 0.42 | 80.03 |
| 4 | 15.16398 | 0.45 | 80.13 |
| 5 | 13.9421 | 0.50 | 80.13 |
| 6 | 10.7532 | 0.52 | 80.27 |
| 7 | 7.07297 | 0.52 | 80.13 |
| 8 | 4.34018 | 0.50 | 80.13 |
| NTC | - | - | - |

**Table S1-H** RRT-PCR results for serially-diluted RNA of N8 subtype

| X (RNA copies=1.95×10^x^) | Mean Ct value | Derivative of DC (≤) | Mean Tm value |
| --- | --- | --- | --- |
| 0 | 31.1986 | 0.33 | 78.83 |
| 1 | 28.5715 | 0.37 | 78.9 |
| 2 | 25.0285 | 0.41 | 79.07 |
| 3 | 26.52977 | 0.44 | 79.2 |
| 4 | 23.63413 | 0.48 | 79.2 |
| 5 | 20.13287 | 0.52 | 79.2 |
| 6 | 16.74847 | 0.55 | 79.3 |
| 7 | 13.369 | 0.55 | 79.2 |
| 8 | 10.18593 | 0.54 | 79.2 |
| NTC | - | - | - |

**Table S1-I** RRT-PCR results for serially-diluted RNA of N9 subtype

| X (RNA copies= 1.874×10^x^) | Mean Ct value | Derivative of DC (≤) | Mean Tm value |
| --- | --- | --- | --- |
| 0 | 27.99207 | 0.34 | 78.03333 |
| 1 | 27.14123 | 0.35 | 78.2 |
| 2 | 26.70493 | 0.38 | 78.32 |
| 3 | 25.5133 | 0.44 | 78.26667 |
| 4 | 22.15473 | 0.5 | 78.26667 |
| 5 | 18.58013 | 0.58 | 78.26667 |
| 6 | 14.9373 | 0.6 | 78.26667 |
| 7 | 11.76027 | 0.6 | 78.26667 |
| 8 | 8.566413 | 0.58 | 78.26667 |
| NTC | - | - | - |
