# Supplementary material for: Neuraminidase Subtyping of Avian Influenza Viruses with PrimerHunter-Designed Primers and Quadruplicate Primer Pools
Source: PLoS One. 2013 Nov 29;8(11):e81842. doi: 10.1371/journal.pone.0081842 (PMC3843705; doi:10.1371/journal.pone.0081842)
Supplement: Table S2 — Result of Real-time PCR with pooled primers for N1 to N9 plasmid. NA plasmids (105 copies) of each NA subtype were used as the templates for Real-time PCR. The primer-pool combination comprises 4 reactions, A, B, C and D, as showed in Table 2. Mean Ct value and Tm value were calculated for the 3 repetition of each reaction. "-" means Tm value of the R-PCR product is too low to be detected or the dissociation curve (DC) is irregular. Positive reactions have Ct≤28 with expected Tm-value range (as shown in Table 1) and dissociation curve. (DOC) [file pone.0081842.s002.doc]

**Table S2. Result of Real-time PCR with pooled primers for N1 to N9 plasmid.**

The results of Real-time PCRs (R-PCRs) using pooled primers and NA plasmids (10^5^ copies) as the templates were shown in Table S2 from A to I. The primer-pool combination comprises 4 reactions, A, B, C and D, as showed in Table 2. For example, A reaction includes primer pairs specific for N2, N6 and N7 genes. Mean Ct value and Tm value were calculated for the 3 repetition of each reaction. "-" means Tm value of the R-PCR product is too low to be detected or the dissociation curve (DC) is irregular. Positive reactions should have Ct≤28 (according to the results of this experiment) with expected Tm-value range (as shown in Table 1) and dissociation curve.

**Table S2-A** Results of N1-plasmid R-PCRs with primer pools

| Reaction | Mean Ct value | Derivative of DC (≤) | Mean Tm value |
| --- | --- | --- | --- |
| A (N2, N6, N7) | 29.71 | 0.16 | 79.10 |
| B (N4, N5, N7, N8) | 30.54 | 0.14 | - |
| C (N3, N5, N9) | 28.46 | 0.24 | 75.90 |
| D (N1, N4, N6, N9) | 18.20 | 0.44 | 78.13 |

**Table S2-B** Results of N2-plasmid R-PCRs with primer pools

| Reaction | Mean Ct value | Derivative of DC (≤) | Mean Tm value |
| --- | --- | --- | --- |
| A (N2, N6, N7) | 15.57 | 0.45 | 77.25 |
| B (N4, N5, N7, N8) | 23.26 | 0.25 | - |
| C (N3, N5, N9) | 28.047 | 0.14 | - |
| D (N1, N4, N6, N9) | 29.57 | 0.14 | - |

**Table S2-C** Results of N3-plasmid R-PCRs with primer pools

| Reaction | Mean Ct value | Derivative of DC (≤) | Mean Tm value |
| --- | --- | --- | --- |
| A (N2, N6, N7) | 29.33 | 0.21 | 78.80 |
| B (N4, N5, N7, N8) | 29.75 | 0.33 | - |
| C (N3, N5, N9) | 19.08 | 0.38 | 76.30 |
| D (N1, N4, N6, N9) | 29.76 | 0.03 | 74.57 |

**Table S2-D** Results of N4-plasmid R-PCRs with primer pools

| Reaction | Mean Ct value | Derivative of DC (≤) | Mean Tm value |
| --- | --- | --- | --- |
| A (N2, N6, N7) | 29.27 | 0.20 | 79.23 |
| B (N4, N5, N7, N8) | 17.76 | 0.67 | 77.00 |
| C (N3, N5, N9) | 27.94 | 0.34 | 76.03 |
| D (N1, N4, N6, N9) | 17.87 | 0.66 | 77.30 |

**Table S2-E** Results of N5-plasmid R-PCRs with primer pools

| Reaction | Mean Ct value | Derivative of DC (≤) | Mean Tm value |
| --- | --- | --- | --- |
| A (N2, N6, N7) | 29.68 | 0.16 | 79.00 |
| B (N4, N5, N7, N8) | 16.28 | 0.65 | 76.50 |
| C (N3, N5, N9) | 16.66 | 0.56 | 76.50 |
| D (N1, N4, N6, N9) | 29.61 | 0.22 | 78.40 |

**Table S2-F** Results of N6-plasmid R-PCRs with primer pools

| Reaction | Mean Ct value | Derivative of DC (≤) | Mean Tm value |
| --- | --- | --- | --- |
| A (N2, N6, N7) | 15.55 | 0.32 | 78.00 |
| B (N4, N5, N7, N8) | 30.09 | 0.14 | 76.50 |
| C (N3, N5, N9) | 31.28 | 0.22 | 75.87 |
| D (N1, N4, N6, N9) | 15.50 | 0.35 | 78.35 |

**Table S2-G** Results of N7-plasmid R-PCRs with primer pools

| Reaction | Mean Ct value | Derivative of DC (≤) | Mean Tm value |
| --- | --- | --- | --- |
| A (N2, N6, N7) | 18.21 | 0.33 | 79.47 |
| B (N4, N5, N7, N8) | 18.08 | 0.35 | 80.00 |
| C (N3, N5, N9) | 28.35 | 0.32 | 76.00 |
| D (N1, N4, N6, N9) | 29.99 | 0.26 | - |

**Table S2-H** Results of N8-plasmid R-PCRs with primer pools

| Reaction | Mean Ct value | Derivative of DC (≤) | Mean Tm value |
| --- | --- | --- | --- |
| A (N2, N6, N7) | 29.03 | 0.22 | 79.33 |
| B (N4, N5, N7, N8) | 17.03 | 0.48 | 79.20 |
| C (N3, N5, N9) | 28.13 | 0.33 | 76.00 |
| D (N1, N4, N6, N9) | 29.33 | 0.28 | - |

**Table S2-I** Results of N9-plasmid R-PCRs with primer pools

| Reaction | Mean Ct value | Derivative of DC (≤) | Mean Tm value |
| --- | --- | --- | --- |
| A (N2, N6, N7) | 29.50 | 0.16 | 79.10 |
| B (N4, N5, N7, N8) | 31.28 | 0.14 | - |
| C (N3, N5, N9) | 17.15 | 0.41 | 78.20 |
| D (N1, N4, N6, N9) | 17.91 | 0.42 | 78.20 |
