# Supplementary material for: Neuraminidase Subtyping of Avian Influenza Viruses with PrimerHunter-Designed Primers and Quadruplicate Primer Pools
Source: PLoS One. 2013 Nov 29;8(11):e81842. doi: 10.1371/journal.pone.0081842 (PMC3843705; doi:10.1371/journal.pone.0081842)
Supplement: Table S3 — Result of Real-time RT-PCR with pooled primers for N1 to N9 RNA. Pooled primers and the RNA templates of each NA subtype extracted from AIV-infected allantoid fluids were used in Real-time RT-PCR. The primer-pool combination comprises 4 reactions (A, B, C and D) as shown in Table 2. Mean Ct value and Tm value were calculated for the 3 repetition of each reaction. The symbol "-" means the Tm value of the R-PCR product is too low to be detected or the dissociation curve (DC) is irregular (e.g. multiple peaks). Positive reactions have Ct≤28 with expected Tm-value range (as shown in Table 1) and dissociation curve. (DOC) [file pone.0081842.s003.doc]

**Table S3. Result of Real-time RT-PCR with pooled primers for N1 to N9 RNA.**

The results of Real-time RT-PCR (RRT-PCR) using pooled primers and the RNA templates of N1 to N9-subtype AIVs extracted from AIV-infected allantoid fluids were shown in Table S3 from A to I. The primer-pool combination comprises 4 reactions (A, B, C and D) as shown in Table 2. A reaction includes primer pairs for N2, N6 and N7 genes, and B reaction includes primer pairs for N4, N5, N7and N8, so on and so forth. Mean Ct value and Tm value were calculated for the 3 repetition of each reaction. The symbol "-" means the Tm value of the R-PCR product is too low to be detected or the dissociation curve (DC) is irregular (e.g. multiple peaks). Positive reactions should have Ct≤28 with expected Tm-value range (as shown in Table 1) and dissociation curve.

**Table 3-A** Results of N1 RRT-PCRs with primer pools

| Reaction | Mean Ct value | Derivative of DC (≤) | Mean Tm value |
| --- | --- | --- | --- |
| A (N2, N6, N7) | 29.58 | 0.2 | 70.70 |
| B (N4, N5, N7, N8) | 28.20 | 0.16 | 69.70 |
| C (N3, N5, N9) | 28.11 | 0.24 | 76.30 |
| D (N1, N4, N6, N9) | 24.16 | 0.4 | 78.30 |

**Table 3-B** Results of N2 RRT-PCRs with primer pools

| Reaction | Mean Ct value | Derivative of DC (≤) | Mean Tm value |
| --- | --- | --- | --- |
| A (N2, N6, N7) | 24.17 | 0.4 | 77.60 |
| B (N4, N5, N7, N8) | 28.36 | 0.18 | 70.30 |
| C (N3, N5, N9) | 25.06 | 0.28 | 76.60 |
| D (N1, N4, N6, N9) | 30.41 | 0.21 | 75.30 |

**Table 3-C** Results of N3 RRT-PCRs with primer pool

| Reaction | Mean Ct value | Derivative of DC (≤) | Mean Tm value |
| --- | --- | --- | --- |
| A (N2, N6, N7) | 28.65 | 0.18 | 71.70 |
| B (N4, N5, N7, N8) | 28.10 | 0.12* | - |
| C (N3, N5, N9) | 10.50 | 0.45 | 76.30 |
| D (N1, N4, N6, N9) | 29.43 | 0.18 | 75.00 |

*irregular curves with 3 peaks

**Table 3-D** Results of N4 RRT-PCRs with primer pools

| Reaction | Mean Ct value | Derivative of DC (≤) | Mean Tm value |
| --- | --- | --- | --- |
| A (N2, N6, N7) | 29.41 | 0.20 | 70.70 |
| B (N4, N5, N7, N8) | 15.05 | 0.45 | 76.90 |
| C (N3, N5, N9) | 25.07 | 0.28* | - |
| D (N1, N4, N6, N9) | 14.73 | 0.61 | 77.30 |

*irregular curves with multiple peaks

**Table 3-E** Results of N5 RRT-PCRs with primer pools

| Reaction | Mean Ct value | Derivative of DC (≤) | Mean Tm value |
| --- | --- | --- | --- |
| A (N2, N6, N7) | 28.92 | 0.17 | 71.40 |
| B (N4, N5, N7, N8) | 17.62 | 0.39 | 76.70 |
| C (N3, N5, N9) | 17.79 | 0.42 | 76.70 |
| D (N1, N4, N6, N9) | 29.79 | 0.24 | 75.30 |

**Table 3-F** Results of N6 RRT-PCRs with primer pools

| Reaction | Mean Ct value | Derivative of DC (≤) | Mean Tm value |
| --- | --- | --- | --- |
| A (N2, N6, N7) | 16.87 | 0.31 | 78.30 |
| B (N4, N5, N7, N8) | 23.56 | 0.17* | 79.50 |
| C (N3, N5, N9) | 25.93 | 0.18 | 73.40 |
| D (N1, N4, N6, N9) | 16.61 | 0.24 | 78.20 |

*irregular curves with 3 peaks

**Table 3-G** Results of N7 RRT-PCRs with primer pools

| Reaction | Mean Ct value | Derivative of DC (≤) | Mean Tm value |
| --- | --- | --- | --- |
| A (N2, N6, N7) | 18.29 | 0.35 | 79.45 |
| B (N4, N5, N7, N8) | 18.31 | 0.37 | 79.45 |
| C (N3, N5, N9) | 24.73 | 0.28 | 76.10 |
| D (N1, N4, N6, N9) | 29.89 | 0.21 | 74.80 |

**Table 3-H** Results of N8 RRT-PCRs with primer pools

| Reaction | Mean Ct value | Derivative of DC (≤) | Mean Tm value |
| --- | --- | --- | --- |
| A (N2, N6, N7) | 28.28 | 0.16 | 71.00 |
| B (N4, N5, N7, N8) | 15.61 | 0.47 | 78.90 |
| C (N3, N5, N9) | 24.92 | 0.29* | 76.30 |
| D (N1, N4, N6, N9) | 28.85 | 0.18 | 74.45 |

*irregular curves with 3 peaks

**Table 3-I** Results of N9 RRT-PCRs with primer pools

| Reaction | Mean Ct value | Derivative of DC (≤) | Mean Tm value |
| --- | --- | --- | --- |
| A (N2, N6, N7) | 24.36 | 0.20 | 75.60 |
| B (N4, N5, N7, N8) | 24.71 | 0.20 | 79.90 |
| C (N3, N5, N9) | 15.46 | 0.34 | 77.60 |
| D (N1, N4, N6, N9) | 14.70 | 0.34 | 77.60 |
