# Supplementary material for: Neuraminidase Subtyping of Avian Influenza Viruses with PrimerHunter-Designed Primers and Quadruplicate Primer Pools
Source: PLoS One. 2013 Nov 29;8(11):e81842. doi: 10.1371/journal.pone.0081842 (PMC3843705; doi:10.1371/journal.pone.0081842)
Supplement: Table S4 — NA subtypes of the 68 AIV swab samples determined in this study. Sixty-eight AIV swab samples from wild birds (ducks, gulls and murres) at St. John’s, Canada were determined for their NA subtypes by RT-PCR with the primer pairs designed in this study. Subsequent amplification and sequencing of longer NA genes were performed, either with other primers, or with the combination of other primers with the NA-subtyping primers in this study, to confirm the accuracy of the NA subtyping methods in this study. The Genbank accession numbers of the NA genes were available for 57 of the 68 samples. *represents samples (23) determined for their NA subtypes by primer-pool RT-PCRs, while other samples (45) were performed individual primer paired RT-PCRs. (DOC) [file pone.0081842.s004.doc]

**Table S4. The NA subtypes of the 68 AIV swab samples determined in this study.**

Sixty-eight AIV swab samples from wild birds (ducks, gulls and murres) at St. John’s, Canada were determined for their NA subtypes by RT-PCR with the primer pairs designed in this study. Amplification of longer NA sequences were tried after the identification of the NA subtypes, either with other primers, or with the combination of other primers with the NA-subtyping primers in this study, The acquired NA genes were purified and sequenced to confirm the accuracy of the NA subtyping methods in this study. The Genbank accession numbers of the NA genes were available for 57 of the 68 samples, the other 8 samples (“-^a^”) only have NA sequences with size less than 300bp (available under request) and could not be accepted by Genbank database, for another 3 samples (“-”) gene sequencing has not been performed yet.

*represents samples (23) determined for their NA subtypes by primer-pool RT-PCRs, while other samples (45) were performed individual primer paired RT-PCRs.

| Avian influenza virus (swab sample) | NA subtype  by RT-PCR | NA gene sequencing | Genbank accession number |
| --- | --- | --- | --- |
| A/American black duck/St. John's/732/2008 (H3N8) | N8* | N8 | -^a^ |
| A/American black duck/St. John’s/734/2008(H3N8) | N8* | N8 | KC492256 |
| A/American black duck/St. John’s/807/2008(H4N4) | N4* | N4 | KC492264 |
| A/American black duck/St. John’s/812/2008(H2N6) | N6* | N6 | KC492272 |
| A/American black duck/St. John’s/819/2008(H4N6) | N6* | N6 | KC492280 |
| A/American black duck/St. John’s/826/2008(H4N6) | N6* | N6 | KC492288 |
| A/American black duck/St. John’s/836/2008(H2N4) | N4* | N4 | KC492296 |
| A/American black duck/St. John’s/840/2008(H2N4) | N4* | N4 | KC492304 |
| A/Domestic duck/St. John’s/MW668/2010(H1N1) | N1* | N1 | KC464560 |
| A/American black duck/ St. John’s/1146/2009(H1N1) | N1* | N1 | KC464568 |
| A/American black duck/ St. John’s/1148/2009(H1N1) | N1* | N1 | KC464576 |
| A/American black duck/ St. John’s/1150/2009(H1N1) | N1* | N1 | KC464584 |
| A/American black duck/ St. John’s/1181/2009(H5N4) | N4* | N4 | KC464592 |
| A/American black duck/St. John’s/MW609/2010(H4N6) | N6* | N6 | KC492312 |
| A/American black duck/St. John’s/MW861/2010(H4N6) | N6* | N6 | KC492320 |
| A/Mallard/St. John’s/PR021/2010(H4N6) | N6* | N6 | KC492328 |
| A/American black duck/St. John’s/MW662/2010(H3N6) | N6* | N6 | KC492336 |
| A/Duck/St. John's/MW721/2010(H6N8) | N8* | N8 | KC492344 |
| A/American black duck/St. John’s/MW733/2010(H6N6) | N6* | N6 | KC492352 |
| A/American black duck/St. John’s/PR007/2010(H6N6) | N6* | N6 | KC492360 |
| A/American black duck/St. John’s/MW774/2010(H11N9) | N9* | N9 | KC492368 |
| A/American black duck/St. John’s/MW819/2010(H11N3) | N3* | N3 | KC492376 |
| A/American black duck/St. John’s/GR252/2011(H3N2) | N2 | N2 | KC492384 |
| A/American black duck/St. John’s/GR256/2011(H3N2) | N2 | N2 | KC492392 |
| A/American black duck/St. John’s/GR396/2011(H3N2) | N2 | N2 | KC492400 |
| A/Mallard/St. John’s/GR475/2011(H2N2) | N2 | N2 | KC492408 |
| A/American black duck/St. John’s/GR490/2011(H3N2) | N2 | N2 | KC492416 |
| A/American black duck/St. John’s/GR679/2011(H3N2) | N2 | N2 | -^a^ |
| A/Northern pintail/St. John’s/GR683/2011(H2N2) | N2 | N2 | KC492438 |
| A/American black duck/St. John’s/GR397/2011(H3N8) | N8 | - | - |
| A/American black duck/St. John’s/GR496/2011(H3N2) | N2 | N2 | -^a^ |
| A/American black duck/St. John’s/GR328/2011(H3N2) | N2 | - | - |
| A/American black duck/St. John’s/GR329/2011(H3N2) | N2 | - | - |
| A/Gull/St. John's/1413/2009(H16N9) | N9* | N9 | KC845029 |
| A/Herring gull/St. John's/YH019/2010(H16N3) | N3 | N3 | KC845122 |
| A/Herring gull/St. John's/YH022/2010(H13N6) | N6 | N6 | -^a^ |
| A/Gull/St. John's/YH038/2010(H13N6) | N6 | N6 | KC845137 |
| A/Herring gull/St. John's/GR032/2010(H16N3) | N3 | N3 | KC845045 |
| A/Great black-backed gull/St. John's/AB001/2011(H9N9) | N9 | N9 | KC845037 |
| A/Herring gull/St. John's/GR366/2011(H16N3) | N3 | N3 | KC845053 |
| A/Ring-billed gull/St. John's/GR384/2011(H13N6) | N6 | N6 | KC845061 |
| A/Herring gull/St. John's/GR530/2011(N6) | N6 | N6 | KC845068 |
| A/Herring gull/St. John's/GR578/2011(H13N6) | N6 | N6 | KC845076 |
| A/Herring gull/St. John's/GR654/2011(H13N6) | N6 | N6 | KC845084 |
| A/Herring gull/St. John's/GR656/2011(H13N6) | N6 | N6 | KC845092 |
| A/Herring gull/St. John's/GR657/2011(H13N6) | N6 | N6 | KC845100 |
| A/Herring gull/St. John's/GR658/2011(H13N6) | N6 | N6 | KC845108 |
| A/Common murre/St. John’s/AB318/2011(H1N2) | N2 | N2 | KC895599 |
| A/Common murre/St. John’s/AB319/2011(H1N2) | N2 | N2 | KC895607 |
| A/Common murre/St. John’s/AB332/2011(H1N2) | N2 | N2 | -^a^ |
| A/Common murre/St. John’s/AB335/2011(H1N2) | N2 | N2 | KC895645 |
| A/Common murre/St. John’s/AB353/2011(H1N2) | N2 | N2 | KC895674 |
| A/Common murre/St. John’s/AB375/2011(H1N2) | N2 | N2 | KC895697 |
| A/Common murre/St. John’s/HM530/2011(H1N2) | N2 | N2 | KC895734 |
| A/Common murre/St. John’s/AB438/2011(H1N2) | N2 | N2 | KC895718 |
| A/Common murre/St. John’s/HM575/2011(H1N2) | N2 | N2 | KC895742 |
| A/Common murre/St. John’s/AB340/2011(H1N2) | N2 | N2 | -^a^ |
| A/Common murre/St. John’s/AB376/2011(H1N2) | N2 | N2 | KC895750 |
| A/Common murre/St. John’s/AB351/2011(H1N2) | N2 | N2 | -^a^ |
| A/Common murre/St. John’s/AB358/2011(H1N2) | N2 | N2 | KC895682 |
| A/Common murre/St. John’s/HM524/2011(H1N2) | N2 | N2 | KC895728 |
| A/Common murre/St. John’s/AB331/2011(H1N2) | N2 | N2 | - ^a^ |
| A/Common murre/St. John’s/AB432/2011(H1N2) | N2 | N2 | KC895710 |
| A/Common murre/St. John’s/AB380/2011(H1N2) | N2 | N2 | KC895703 |
| A/Common murre/St. John’s/AB324/2011(H1N2) | N2 | N2 | KC895615 |
| A/Common murre/St. John’s/AB327/2011(H1N2) | N2 | N2 | KC895623 |
| A/Common murre/St. John’s/AB341/2011(H1N2) | N2 | N2 | KC895659 |
| A/Common murre/St. John’s/AB364/2011(H1N2) | N2 | N2 | KC895689 |
