# Supplementary material for: Neuraminidase Subtyping of Avian Influenza Viruses with PrimerHunter-Designed Primers and Quadruplicate Primer Pools
Source: PLoS One. 2013 Nov 29;8(11):e81842. doi: 10.1371/journal.pone.0081842 (PMC3843705; doi:10.1371/journal.pone.0081842)
Supplement: Figure S1 — Gel electrophoresis image of one-step RT-PCR with pooled primers. The electrophoresis was run with 1.5% agarose gel. A, B, C and D represent pooled-primer reaction A to D, as showed in Table 2. M represents 100bp DNA marker (NEB, USA). The NA-subtyping results for the 5 swab samples were further confirmed by gene sequencing and related genes were submitted to the Genbank database under accession numbers KC464568, KC492344, KC464592, KC492256, and KC492368. (DOC) [file pone.0081842.s005.doc]

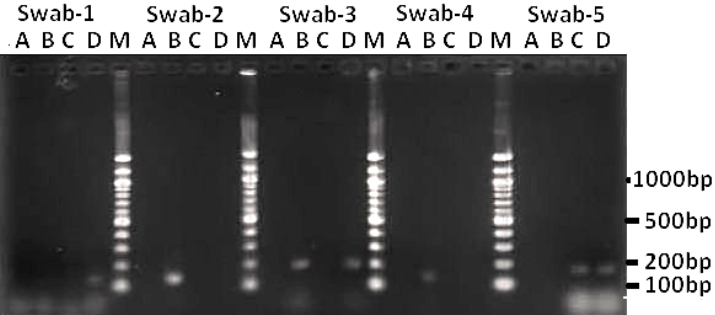


**Figure S1. Gel electrophoresis image of one-step RT-PCR with pooled primers.** The electrophoresis was run with 1.5% agarose gel. A, B, C and D represent pooled-primer reaction A to D, as showed in Table 2. M represents 100bp DNA marker (NEB, USA). The NA-subtyping results for the 5 swab samples were confirmed by gene sequencing and related genes were submitted to the Genbank database. The NA gene of Swab-1, *A/American black duck/St. John’s/1146/2009(H1N1)*, was under Genbank accession number KC464568. The NA gene of Swab 2, *A/Duck/St. John's/MW721/2010(H6N8)*, was under Genbank accession number KC492344. The NA gene of Swab 3, *A/American black duck/ St. John’s/1181/2009(H5N4)*, was under Genbank accession number KC464592. The NA gene of Swab 4, A*/American black duck/St. John’s/734/2008(H3N8*, was under Genbank accession number KC492256. The NA gene of Swab 5, *A/American black duck/St. John’s/MW774/2010(H11N9)*, was under Genbank accession number KC492368.
