# Supplementary material for: Neuraminidase Subtyping of Avian Influenza Viruses with PrimerHunter-Designed Primers and Quadruplicate Primer Pools
Source: PLoS One. 2013 Nov 29;8(11):e81842. doi: 10.1371/journal.pone.0081842 (PMC3843705; doi:10.1371/journal.pone.0081842)
Supplement: Figure S2 — Result of Real-time RT-PCR with pooled primers and N4 RNA template. RNA was extracted from H8N4 AIV-infected allantoid fluids. The amplification curves (part A) showed positive amplification in B and D reactions of the primer-pooled Real-time RT-PCR, while negatives in A and C reactions. The dissociation curves (part B) of B and D reactions are distinct from those of A and C reactions. (DOCX) [file pone.0081842.s006.docx]

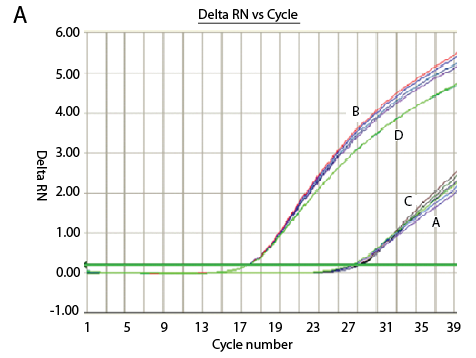


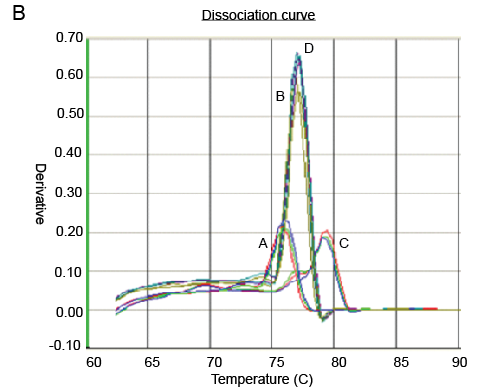


**Figure S2. Result of Real-time RT-PCR with pooled primers and N4 RNA template.** RNA was extracted from H8N4 AIV-infected allantoid fluids. The amplification curves (part A) showed positive amplification in B and D reactions of the primer-pooled real-time RT-PCR, while negatives in A and C reactions. The dissociation curves (part B) of B and D reactions are distinct from those of A and C reactions.
